# Muslim Immigrant Men's and Women's Attitudes Towards Intimate Partner Violence

**DOI:** 10.5964/ejop.v13i4.1411

**Published:** 2017-11-30

**Authors:** Marialuisa Gennari, Cristina Giuliani, Monica Accordini

**Affiliations:** a Department of Psychology, Università Cattolica del Sacro Cuore, Brescia, Italy; b Department of Psychology, Università Cattolica del Sacro Cuore, Milano, Italy; Webster University Geneva, Geneva, Switzerland; University of Liverpool, Liverpool, United Kingdom

**Keywords:** IPV, Muslim immigrants, male and female attitudes, migration risk factors

## Abstract

This study aims to study the attitudes towards Intimate Partner Violence (IPV) in a group of Muslim immigrants. To this end, six focus-groups were conducted involving 42 first-generation Muslim immigrants (21 males and 21 females) from Pakistan, Egypt and Morocco. Focus groups transcripts were then analyzed using the software ATLAS.ti. Irrespectively of nationality, couples replicate relational models learnt in their country of origin, implying a rigid gender-based role division. Women are considered less socially competent if compared to men and therefore in need of protection. Divorce is possible only in case of severe danger: women have to stand beside their husbands and maintain family unity. Even though they are not directly related to IPV, these factors may be key in determining its onset and perpetration. With regards to ethnic background, Pakistani interviewees not only seem to acknowledge the possible occurrence of violence within couple relationships, they also accept it as a mean to regulate socially dysfunctional behaviors. Both Moroccan males and females denounce the impact of post migration stressors as potential triggers of IPV. The distance from one’s family of origin in migration is perceived as problematic by both men and women, however, while males’ distance from their kin might make them feel overwhelmed with family responsibilities and give way to deviant behaviors, women suffer from the lack of support and protection by their extended family. Implications for practice are also discussed.

Over the past decades, the issue of domestic violence against women within immigrant families has become a major research concern. As shown by an increasing body of research, immigrant and refugee couples are particularly vulnerable to domestic violence: immigrant women report both a higher frequency of intimate partner violence (IPV) episodes (ranging from 30% to 60%) as well as a greater severity of abusive incidents if compared to the general population living in high income Western countries (Hass, Ammar, & Orloff, 2006; Jasinski & Kantor, 2001; Raj & Silverman, 2003; Vatnar & Bjørkly, 2010).

The rationale for such a phenomenon has been attributed to the various dimensions underlined in the literature as contributing to domestic violence as well as to the many challenges posit by the post migration context (Capaldi, Knoble, Shortt, & Kim, 2012; Giuliani & Gennari, 2014), including the socio-cultural variability of the various migration countries (Gozzoli, 2016; Tamanza, Gozzoli, & Gennari, 2016). Several studies have shown that socio-cultural variables (e.g., attitudes, cultural beliefs, and values) are critical factors leading to an increased tolerance towards domestic violence (Haj-Yahia, 2000, 2003; Murnen, Wright, & Kaluzny, 2002). If such values and attitudes are retained also in the hosting country, they contribute to the perpetration of abusive conducts and prevent women from seeking help (Vives-Cases et al., 2013).

More specifically, immigrant couples coming from majority-Muslim countries show higher IPV rates if compared to their non-immigrant counterparts: for example, Moroccan immigrant women in Spain (Colorado-Yohar et al., 2012), Iraqi (Barkho, Fakhouri, & Arnetz, 2011), as well as Pakistani and South-Asian (Adam & Schewe, 2007; Lee & Hadeed, 2009) women immigrated to the U.S. report being exposed to abusive behaviors to a greater extend if compared to the native population and even to other non-immigrant Muslim women.

## Muslim Immigrants’ Attitudes Towards IPV

Scientific literature exploring the relationship between Muslim immigrants attitudes and IPV has especially focused on three, deeply interrelated, key themes affecting the emergence and perpetuation of spousal violence and the help-seeking behaviors among immigrants:

- *Traditional gender role attitudes*. Traditional gender role attitudes have been found to facilitate violence and to provide cultural justification for abusive behaviors (Bhanot & Senn, 2007) while encouraging self-blaming attitudes in abused women (Abu-Ras, 2007). Both Muslim men and women have the duty to preserve the honor of their family, however, they are expected to show different behaviors: men should be authoritative and courageous while women are encouraged to be submissive (Kulwicki, 2002). Such gender specific differences imply that men are in control and entitled to exert authority over their wives. In patriarchal cultures, men are socially—and often times legally—responsible for the women in their family, this gives them the right to educate and discipline them (Shalabi, Mitchell, & Andersson, 2015). When a female fails to meet societal expectations or to perform her duties, violence is reckoned as a valid alternative to educate her. Many states in the Muslim world adopt laws and regulations enforcing patriarchal values that give men the responsibility to decide for their female family members while cultural attitudes regarding the social acceptance of violence are widespread (Lee & Hadeed, 2009) and may also be legitimized by a literal interpretation of the Qur’an. This results in different degrees of acceptance of violent behaviors and their normalization in social and intimate relationships: violence may be considered a normative, not deviant, behavior to discipline dependent family members (Ibrahim & Abdalla, 2010).
- *Family integrity and honor*. Family is the key social unit in Muslim societies and family cohesion and honor are regarded as extremely important values. The concepts of shame (*ayb*) and honor (*sharaf*) are at the core of the family structure, therefore all members are called to protect family integrity and respectability (Ammar, 2007). In this view, family needs should precede individual needs and women in particular are the ones who have the responsibility of keeping the family together, maintaining harmony, solidarity and loyalty between members (Abu-Ras, 2007). Privacy and the family integrity should be preserved, even at the sake of the happiness of their individual members. The utmost importance of privacy and honor can prevent abused immigrant women from seeking help for fear of being judged or even stigmatized by friends and relatives. On a similar note, divorce is disregarded and considered to put shame on the family, therefore spouses are advised to stay together even if they are unhappy with their marriage. Women wishing to divorce their husbands (a practice called *khula*) are generally stigmatized and socially disapproved (Levitt & Ware, 2006).
- *Social isolation in the post-migration context*. Although many immigrants experience social isolation, Muslim immigrant women are particularly subject to this experience. When women migrate to reunite to their husbands, they don’t benefit from the protection of their patrilineal kin and become therefore completely dependent on their husbands. Muslim women have been reported to suffer from a double isolation as their husbands often attempt to further isolate them from social life for fear of their becoming too westernized or out of jealousy (Abraham, 2000a; Giuliani & Tagliabue, 2015). The lack of support from their community of origin and the stigma and shame associated to services and shelters are other important elements that might prevent women from adopting help seeking behaviors (Cainkar & Del Toro, 2010).

In light of the literature findings outlined above and considering the lack of studies on immigrant male and female attitudes towards domestic violence (Macey, 1999), the present study aims to explore men’s and women’s attitudes towards IPV in three ethnic groups of Muslims immigrated to Italy from Morocco, Egypt and Pakistan as well as to explore the underlying beliefs and values facilitating or justifying violent behaviors, also in light of the struggles posit by the post-migration context.

In particular, our study aims at addressing the following objectives: 1. investigate the immigrant men and women attitudes with regards to a phenomenon, such as that of domestic violence, that is described a particularly urgent by the scientific literature on the topic; 2. outline the differences between men and women with regards to their attitudes towards domestic violence; 3. outline the differences between the three ethnic groups considered with regards to their attitudes towards domestic violence.

## Method

### Study Design

This paper is an exploratory study employing a cross-sectional qualitative design. In particular, a focus group design was used in order to facilitate interaction and exchange between members and to elicit the emergence of cultural values and norms regarding IPV among Muslim immigrant communities.

Focus groups have been found to be sensitive to cultural issues and particularly useful to address attitudes and beliefs towards sensitive topics (Kitzinger, 1995) as they provide insights into participants’ cultural models and perceptions. Focus groups allow participants to openly discuss their views and to gain a greater awareness of both their own as well as the others’ perspectives (Morgan, 1997). Moreover, this methodology may help participants compare their own situation or viewpoint with that of the community or group they belong to and this is especially important when discussing sensitive topics such IPV. IPV is frequently regarded as a private matter and having the chance to discuss it with others may reduce isolation and social stigma as well as help people gain awareness regarding the social and cultural barriers to help seeking (Yoshihama, 2005).

### Participants

Six focus-groups were conducted from September 2014 to March 2015 with 42 first generation Muslim immigrants (21 M, 21 F), equally divided across the three nations of origin. The six groups were homogeneous for nationality and gender.

Immigrants from Morocco, Egypt and Pakistan were identified for our study and chosen for their representativeness within the Italian context. The 524.775 Moroccans, 135.284 Egyptians and 106.485 Pakistanis officially residing in Italy on January 1, 2014 are amongst the largest minorities in the country. Moreover, it is noteworthy to mention that Pakistanis are among the fastest growing immigrant groups in Italy (Istituto italiano di statistica [ISTAT], 2014).

The 42 participants lived in Northern Italy and were recruited through a snowball sampling method according the following criteria: (1) being first-generation Muslim immigrants and being above 18 years of age; (2) coming from Morocco, Egypt or Pakistan; (3) being married and having lived in Italy with one’s family (partner and child(ren)) for at least two years. Participants were screened for age, education, employment status and length of stay in Italy, these data are reported in Table 1.

**Table 1:** Participants’ Socio-Demographic Characteristics

Characteristic	Men				Women			
	Morocco (*n* = 7)	Egypt (*n* = 7)	Pakistan (*n* = 7)	Total (*n* = 21)	Morocco (*n* = 7)	Egypt (*n* = 7)	Pakistan (*n* = 7)	Total (*n* = 21)
Age								
Median	35.00	32.00	29.00	32.00	30.00	33.00	31.00	31.00
Mean	34.71	34.00	31.71	33.48	30.57	33.85	32.14	32.24
Standard deviation	7.04	8.52	6.82	7.20	4.76	9.92	4.98	6.71
Residence years in Italy								
Median	13.00	11.00	8.00	11.00	9.00	10.00	6.00	8.00
Mean	13.57	11.00	10.00	11.52	10.14	11.85	5.85	9.29
Standard deviation	6.32	7.79	4.32	6.17	4.63	8.49	2.96	6.12
Education *(frequencies)*								
No schooling	3	1	2	6	1	0	3	4
Up to 10 years	4	5	5	14	6	4	4	14
>11 years	0	1	0	1	0	3	0	3
Employment status *(frequencies)*								
Unemployed	3	0	0	3	0	0	0	0
Housewife	0	0	0	0	5	7	7	19
Unskilled worker	4	5	7	16	2	0	0	2
Small businessman	0	2	0	2	0	0	0	0

### Procedure and Data Collection

Several language schools, ethnic, and religious associations were contacted to recruit the first participants who were then asked to inform their fellow nationals about the possibility to take part in the research project. As mentioned above, 42 participants met the inclusion criteria. Selected participants were explained the aims of the research and invited to sign the consent form. After having been granted anonymity, interviewees filled in a short questionnaire with their background information. Subsequently they were invited to take part in a 90 minutes focus group discussion after which they were rewarded with a 10 euro gift voucher for shopping at local market. Focus groups took place in private homes or in public places (libraries, schools, local associations) and were led by two Italian experienced moderators, a male and a female, as suggested by several studies (e.g., Krueger & Casey, 2000). In particular, female groups were moderated by a female conductor, with the male researcher mainly assisting with field notes and observation while the contrary happened with male groups. For the scopes of this research, an experienced moderator was defined as someone having conducted at least 10 focus groups and having a specific expertise with working with immigrant populations, particularly those coming from majority Muslim countries.

Moderators used a semi-structured focus-group guide containing a set of questions and statements to explore the topic of IPV among immigrant Muslim couples. Questions focused both on the factors that, according to the literature, may contribute to cause or perpetrate domestic violence as well as on domestic violence per se. Among the precipitating factors, participants were asked about the challenges and hardships faced in the new country, their relationships with their family and community as well as the main differences between being in a couple in Italy and back in their home country.

Questions and prompts used to investigate domestic violence are based on the literature on IPV and have been used elsewhere in focus group contexts (Beaulaurier, Seff, & Newman, 2008; Petersen, Moracco, Goldstein, & Clark, 2003). Prompts were intentionally vague so to stimulate the discussion about sensitive issues without suggesting any desired answer. Questions discussed by participants included: “What does the words intimate partner violence tell you?”; “What happens when couples fight? How are fights handled?”; “What happens when arguments or fights get “out of hand”?; “How are marital conflicts handled in your own community? Is there any difference between what happens in your home country and what happens here in Italy?”.

In order to further reduce the risk of socially desirable responses, the following short vignette describing a conflictual couple relationship was also included along with the above mentioned prompts and participants were asked to comment on the situation: “Besma is 36; together with their children, she joined her husband three years ago. Now the couple is experiencing serious problems, they don’t get along and he mistreats her. Besma feels unhappy and doesn’t know what to do…”.

The research was granted approval by the University Ethical Committee, which fulfilled ethical standards of the Italian Psychology Association (AIP, Associazione Italiana di Psicologia).

### Data Analysis

Focus groups were recorded and transcribed verbatim in order to perform a content analysis of the transcripts. Content analysis can be a useful method for organizing and summarizing data (Berg, 2004) as it allows a more immediate understanding of the text as it identifies the underlying themes summarizing the transcripts. Being a data-driven method (Sandelowski, 2000), content analysis allows to draw phenomenological insights directly based on the words and phrases that participants used to voice their attitudes and thoughts.

Content analysis was conducted utilizing Atlas.ti 7.0 (Muhr, 2004), the focus group transcripts were uploaded to the database and then independently coded by two researchers. External team members who were not directly involved with the analysis reviewed the transcripts, methodology and analytic strategy to increase credibility and validity.

The coding procedure followed various steps: initially, two researchers independently coded all the transcripts. Both open and axial coding were used, axial codes were derived from a review of the literature, while open codes were created deductively from the text transcripts (Strauss & Corbin, 1998). After this initial set of coding, the two researchers met to share their individual coding schemes until a unified coding scheme was created. At the end of this second step, researchers analyzed all the six focus groups transcripts again according to the so found set of codes. After the coding was completed, researchers leveraged on the ATLAS.ti co-occurrence frequency table to identify related concepts and create broader codes families (Muhr, 2004). Finally, periodic research meetings involving the whole research group (e.g., the two coders as well as the focus group moderators) in which the published literature, text transcripts and emergent recurrent patterns were discussed in relation to each another served to further refine the list of codes families. Triangulation of data was assured by the presence of colleagues with expertise in both migration issues and qualitative methods (Crabtree & Miller, 1999).

One of the main advantages of using ATLAS.ti it is that it also creates a list of quotations connected to each code and code family, this allows results to be data-driven and prevent researchers from making undue assumptions. Although findings from this procedure may lack generalizability, they are true to the participants’ voice and opinion (Corbin & Strauss, 1990).

## Results

The content analysis results generated 12 codes (called ‘categories’), which can be further clustered into four overarching codes families (called ‘themes’) (see Table 2), each referring to a specific domain.

**Table 2:** Themes and Categories Emerged From the Focus-Groups

Theme	Categories
Attitudes towards violence	Violence acceptance Male duty to control vulnerable women
Cultural norms towards the marital couple bond	Husbands’ supremacy Couple and family unity as a priority Disapproval/unacceptability of divorce Family as origin of the marital contract Families of origin as preferred help-seeking source in case of marital crisis
Impact of the geographical distance from the family of origin	Male loneliness and feeling overwhelmed Men exposure to deviant behaviors Women lack of protection
Women social relations in post-migration context	Women isolation Women employment

### Theme 1: Attitudes Towards Violence

The first theme, made of two categories, refers to views and attitudes concerning violence episodes that can occur in community and family relationships, while no specific mention to the couple bond is made.

#### Violence Acceptance

The first category concerns the normalization of violence within the social context: only people coming from Pakistan report habitual violence episodes in their social relationships. In particular, participants justify violent behaviors as a way to correct or eliminate misconduct according to a set of cultural norms and habits that regulate every aspect of the individuals’ social and private life, indicating appropriate and inappropriate behaviors. A certain degree of violence is therefore considered as a sign of showing interest and care towards someone. Both male and female interviewees seem to share this view, as confirmed by the following quotes:

(Pakistani man2): …in our country if someone is wrong the others need to correct him/her. (Pakistani man1): Yes, they use force sometimes… to correct. (Interviewer): When you say others who are you talking about? The family or the community? (Pakistani man2): The family but also the community around them.

(Pakistani woman): I like it here, kids go to school and teachers do not beat them. In Pakistan each class is composed of 50/55 pupils and things can get very messy so they need to discipline children and most of them are beaten by their teachers…

Conversely, it is important to notice that neither Moroccan nor Egyptian participants mention the legitimation of violent behaviors or explicitly refer to the possibility of using violence in social relationships.

#### Male Duty to Control Vulnerable Women

Another category that Pakistani men only bring about to support violent behaviors against women is connected to the need for social control. It is a specific male duty to control and take care of the female members of the family, especially when they are out of home. Women are in fact considered less socially competent and capable of dealing with the outside world.

(Pakistani man2): Here you are constantly worried about that problem…you are not calm, there is no (Pakistani man1): We live in very numerous, united families, with many siblings and cousins. He wanted to say that back in Pakistan we don’t have this kind of problem, here, on the contrary, we do. Trust me, we have that over there. … you can be calm, there’s always going to be someone watching out in case something happens (Pakistani man3): When you are there there’s people you can trust, you are calm because you know that somebody will always watch out in case something happens…someone who controls your wife and kids. (Pakistani man4): Here it is not as if you were there, in Pakistan. Over there a man can remain calm because women stay among themselves and don’t get into troubles. You feel safe because they talk among themselves, understand and learn things.

### Theme 2: Cultural Norms Towards the Marital Couple Bond

The second theme, made of five categories, concerns the participants’ representations of marriage. As we will see later in the text, those representations are strictly connected to and facilitate couple violence.

#### Husbands’ Supremacy

According to all the participants, irrespectively of their ethnic background, Muslim couples are characterized by a rigid role division and a great power imbalance between men and women. While women prove to be good wives showing devotion and submission to their husbands, it’s a husband’s duty to exert his power and authority. Both male and female interviewees agree that men should occupy a superior and privileged position over women and males’ decisions and wishes should come first. According to this view, women are called to stand beside their husbands, no matter what. Family is the only place where women can find fulfillment. It is also interesting to notice that such a view is promoted by elderly women who try to educate young brides.

(Moroccan man): Moms always recommend their daughters to be patient…they say: “You need to be patient. Women need to put up with their husbands and be tolerant and do as they are asked”.

(Pakistani woman): Women need to follow their husbands’ advice, they have to show respect and obedience because men know better, they know what is best for their wives.

(Egyptian woman): Egyptian men do whatever they want, they don’t need to ask for permission. Women, on the contrary, ask men for the permission to do certain things.

The women who took part in our research agree that self-determination is based upon gender: women should obey and be subdued to their men, while husbands have a much wider array of things they can do or ask for.

(Egyptian woman): I wanted to say that men have the power and control because they are the ones who bring money home so they run the family and decide.

(Pakistani woman): Men and women are at the same level here in Italy. Even though also over there they have the same rights, it’s not the same because women are dependent on their husband.

(Moroccan woman): A good wife shows respect towards her husband and so she has to swallow some bitter pills sometimes, even if she doesn’t agree, she has to be silent and accept.

#### Couple and Family Unity as a Priority

An extremely relevant category raised in particular by female participants has to do with the key value and indissolubility of family bonds. The importance of family ties justifies women’s tolerance of marital conflicts and their acceptance of violent behaviors perpetrated by their spouses.

(Egyptian woman): I would like to have a family and become a mother. Family is the most important thing, especially for a woman, she is the one who has the duty to keep the family together.

(Moroccan woman): If you are experiencing a problem with your husband, you don’t tell anybody. …I don’t want this to happen, especially after having had kids, I’d like my family to be unite, I don’t want anyone to suffer because of me.

(Pakistani woman): My husband came here…so when he came to Italy I had no choice but to follow him and move here too, wives have to follow their men.

#### Disapproval/Unacceptability of Divorce

According to some widespread cultural norms, couples should find a way to remain together in spite of conflicts, misunderstandings and even violence. Women are unlikely to leave their husbands as their family and children come before the individual and couple well-being. Disapproval of divorce is another category emerged from the focus groups, as shown by the following quotes.

(Pakistani woman): Here if you happen to fight with your husband, you want a divorce. …this is what I see happening. On the contrary, in our culture we try to get along and remain together until the end, despite the problems.

(Moroccan woman): As a wife, I’ve always tried to carry on. You know, even when violence-related problems occur, women need to think to their family and their children first.

(Egyptian woman): You keep on going on for the sake of your parents and for your children, this is our duty. Even if there are things you don’t like, you have to accept them, you cannot disrupt your family and have your children go through that.

According to all participants, divorce is rarely a viable option. It needs to be said that while Pakistanis are extremely reluctant to even consider the possibility of divorce, Egyptians and Moroccans seem to be more open.

(Moroccan man): If no other solution is possible there is divorce. … divorce is really the last option but if the spouses don’t get along anymore and they cannot find any solution they have to separate, it’s sad but it’s best for everyone. (Interviewer): Is it possible to get separated in Pakistan? (Pakistani woman): Yes, but it’s extremely unlikely, almost nobody does it. Women think about their children first, they know that have to make any effort for them. It is hard to separate if you have kids because kids will then experience many problems.

#### Family as Origin of the Marital Contract

The occurrence of arranged marriages is another important issue that affects the couple’s life. In these cases, families of origin enter an agreement while spouses cannot express their will or feelings. While men did not comment on this event, interviewed women reported an increased risk of domestic violence in cases of arranged marriages. In these cases spouses don’t know each other before the wedding and this may result in a greater likelihood of misunderstandings, disagreements and relational mismatches.

(Pakistani woman): Here girls are free to choose their future husbands, but in our country parents are the ones who make this choice. …they ask you beforehand, they will ask you: “This is your boyfriend, do you like him or not?” I can’t say I love someone because I’ve never seen him but if my parents ask me: “Do you like him?” and my answer is no, they are going to look for someone else, this is not a big deal.

(Moroccan woman): I’ve seen many, many cases of battered women because in our country arranged marriages outnumber love marriages. When there is an arranged marriage, you don’t know who your husband is going to be and there’s a chance he is not going to be good to you.

#### Families of Origin as Preferred Help-Seeking Source in Case of Marital Crisis

Irrespectively of ethnicity, all participants agree that families of origin are called to help young couples in case of severe marital conflict. Parents in particular mediate couple disputes and lead new couples throughout complex decision-making processes. Therefore, their advice becomes a non-written law to abide to. In order to fully understand this aspect, it needs to be remembered that, as said before, in many cases families of origin highly contributed to the couple formation.

(Moroccan man): I think it’s better if you have a mediator…I’ve gone through this already, you end up fighting constantly. The police is called and you lose your mind and you come to blows. So this is my advice: if I have a problem in my relationship, I call our parents. That’s it! We go there, we talk and we find a solution.

(Egyptian man): So in my opinion it is very good to get help from your family and when there’s a problem between husband and wife and they don’t get any help then they separate.

(Moroccan woman): When the husband starts beating his wife she would go to her parents for help: she would stay at her parents’, this is how it works in our country. …I saw it happen many times.

(Pakistani man): Talking to your parents and elder relatives really helps things go smoother because they give advice and wish the best for everybody. When two people get married, the whole family is involved. Let’s say that something happens between us and we fight, the whole family will intervene: “What problems do you have? Who’s right? Who’s wrong?” So if there’s a problem, they are involved too.

### Theme 3: Impact of the Geographical Distance From the Family of Origin

The third theme, made of three categories, collects topics regarding the impact of losing connection to one’s family of origin. The forced distance from their families, puts couple stability at stake and all interviewees reported feelings of loss and being overwhelmed by an emotional burden they can hardly bear.

#### Male Loneliness and Feeling Overwhelmed

In some cases male participants reported problems connected to feeling overwhelmed with the responsibilities of running a family. Deprived of the protective and surveillance function usually performed by community and extended family members, men or young male immigrants are left alone to bear this responsibility. Male interviewees often report feeling worried and concerned and thus incrementing surveillance efforts over women. Being away for work all day, men experience an increased sense of helplessness and frailty because they cannot be next to their wives and monitor their behaviors:

(Moroccan man): The problem here in Italy is that husbands are busy, they work all day while women don’t do anything, they just take care of the kids. So the greatest problem is that we are away from home while back in Morocco this was not a big deal.

(Egyptian man): Here you have everything you want but in spite of that, we all want to go back home and stay with our wives and children because here they are alone and don’t know anybody…so we are worried.

(Pakistani man): I am alone in taking care of my family and if I have a problem at home it’s a mess…who can I trust? Who can I turn to for help? You see, it is a huge trouble for us, we are constantly worried, we are afraid for our wives and children.

#### Men Exposure to Deviant Behaviors

According to the Moroccan participants’ narrations, immigrant men behavior is deeply affected by the distance from their families of origin. Free from the restrictions and limitations imposed by their older family members and because of the power imbalance within the marital relationship, men feel legitimated to carry out behaviors of any kind, including abusing drugs and committing crimes. Moreover, women, subjugated to their husbands and virtually ignored as potential interlocutors, are in no position to contain such behaviors. In particular, some Moroccan men experience an estrangement from their family life after migration to the point that their conduct betrays the model imposed to a good Muslim father. Several Moroccan male participants reported having witnessed irresponsible and even risky behaviors carried out by their fellow nationals, who seemed oblivious of the damage they were causing to their families. Such a change in life style often results in arguments and strong disagreements within the marital couple. Conflicts may hesitate in violent behaviors (Bélanger & Brisebois, 2010), especially in cases of men drinking to excess, abusing drugs or being involved in illegal activities. According to our interviewees, however, drug abuse is related to, although not causing, violence: both occur due to unemployment and the lack of economic resources.

(Moroccan woman): When they lose their job because they hurt themselves or because their contract has expired…sometimes men ask women for money to go to the bar or buy drugs. As long as women do not complain and obey their husbands everything’s fine but if they say: “I can’t take it anymore!”, then violence begins.

(Moroccan man): A man who always stays at the bar will inevitably have problems with his wife. Some men are seldom at home with their families and when they are home they are drunk because they don’t have money or because they’ve lost their job…so they may come to blows with their wives.

#### Women Lack of Protection

According to all female participants, couple violence results from losing contact with one’s family network. After migration, the kin loses the protective and conflict resolution role it once had over the woman and the couple in general. In particular, women not only report feeling lonely in face of emotional and relational strains, but they also lack protection from the possible abuses perpetrated by their husbands. Therefore, living in Italy for Muslim wives may not only mean coming to terms with an increased emotional distress but also putting their physical safety at stake. Hence, women run the risk of being exposed to an invisible, silent violence, hidden within the comfort of their house’s walls.

(Moroccan woman): This is what I’ve…I’ve come to understand: these men see that women are far away from their families and so they do as they wish in that they exploit them. Back in Morocco women have their families to protect them.

(Pakistani woman): Wives find it very hard to adapt alone, they are always home alone and depend on their husbands for everything…so it depends on how the husband is because she doesn’t have anybody to turn to if something bad happens to her.

### Theme 4: Women Social Relations in Post-Migration Context

The fourth theme, made of two categories, focuses on the attitudes towards the challenges that are inevitably connected to migration. With regards to this category, interviewees underlined both the social isolation women—especially those coming from Pakistan—are confronted with after migration as well as the difficulty in accepting female work, even in face of economic strains.

#### Women Isolation

Pakistanis often report being concerned with the emotional difficulties experienced by their wives due to both the absence of their social network as well as the isolation resulting from their lifestyle. Loneliness and marginalization are the main features of female immigration: locked in their homes, Pakistani women are strangers within a culture they do not belong or participate to, immersed in a language they do not understand and do not learn. In this respect, men underline that many times, even several years after the migration, the encounter between Pakistani women and the Italian community fails to occur, to the point that they will always feel strangers and constantly dream of returning home.

(Pakistani woman): My roots are back there, it’s hard for me to live here. I am always at home by myself and have no-one to talk to, I feel lonely and homesick and I often think about Pakistan, how is it over there, what people are doing.

(Pakistani man): Women are always home, they never go out. They cannot go around. They have to wait ’til Saturday if they need to go to the market. …they have to wait their husbands because they either don’t speak Italian or don’t have the driving license.

Frail and emotionally unstable because of this situation, Pakistani women pour their discontent—which men are unable to handle—on their families.

(Pakistani man): My wife told me she misses her parents and her siblings…I hope this will eventually go away because she is sad and always crying. …women are always sad because they would like to go back there [to Pakistan]…so the family lacks happiness and joy.

Moroccan men are also aware of the social isolation affecting women, however they do not ascribe this situation to migration. They rather call upon economic problems, poverty or the poorly designed political actions undertaken by the Italian government that do not favor integration. Moreover, Moroccan men reckon that, having more time to dedicate to the development of social relations, women have greater chances of socialization in the host country.

(Moroccan man1): I see women in public gardens and foreign women talk to Italian women…it happens…while men find it harder to talk with others if they are not colleagues. I mean, now women have far more chances to integrate than men. (Moroccan man2): It may happen that our wives here cannot go around because they don’t have a car or a driving license…but these are economic problems, it has nothing to do with our culture or religion. …the European society doesn’t favor women’s integration into society.

#### Women Employment

Another relevant aspect that might put immigrant couples’ stability at stake is the issue of female employment. Unlike the Pakistani and Egyptian immigrants in our sample, who do not want their wives to work, Moroccan men report mixed feelings about this issue. Some, allow their wives to work or attend training courses. Many others, on the contrary, approve of female employment only in case of severe economic hardships, especially if this implies working in close contact with other men.

(Moroccan man): This is good because your wife helps you and then, how can I say? If she has the chance to attend a training course…according to her education and skills…maybe do some training or something.

(Moroccan man): If a woman is happy staying at home and her parents don’t need her help she can do without working, she can stay home which is much better…if a woman, needs something, she has the right to help her parents if they’re in need.

Also when needed, women employment is not always welcomed in Moroccan families: working in mixed environments together with other adult males or working at night may result in arguments and conflicts and may as well lead to the explosion of violence.

(Moroccan man): Maybe women are willing to work because they need to support their families of origin. Husbands don’t agree because women will need to work full-time or do a job they don’t approve of. …these are the actual problems that may lead to violent behaviors. It might be that a woman wants to work in a restaurant and her husband says: “No, you can’t work in a restaurant because I don’t want you to always be surrounded by other men”.

## Discussion

The present paper aims at investigating the attitudes towards domestic violence in a group of immigrants coming from Morocco, Egypt and Pakistan. In particular, our aim was threefold: 1. to investigate the immigrant men and women attitudes with regards to a phenomenon, such as that of domestic violence, that is described a particularly urgent by the scientific literature on the topic; 2. to study the differences between men and women with regards to their perceptions of the topic; and 3. to study the differences between the three ethnic groups considered.

Irrespectively of the interviewees’ gender and nationality, the focus groups transcripts allowed to identify some recurrent topics that might help researchers understand the marital context in which spousal violence maybe perpetrated. In particular, these common topics are:

1. *husband’s supremacy* which also implies women occupy an inferior and subjugated position within the couple and determines a rigid gender-based role division. As already evidenced by the literature on the topic, Muslim immigrants tend to replicate the same traditional gender role patterns adopted in their countries of origin also after migration. In this respect, some authors (Ali, Karamali, & Malik, 2014) have underlined how traditionalism or perpetuation of traditions may lead to family problems due to the immigrants’ poor adaptation to the hosting country;
2. *the women’s duty to keep the couple and family unity as a priority*. Consistently with the literature on the cultural norms regulating family interactions and gender role expectations in Muslim communities (Adam & Schewe, 2007; Ammar, 2007), our findings suggest that women are called to preserve family integrity and cohesion, even in cases when their own happiness and physical safety is at stake;
3. *the disapproval/unacceptability of divorce,* both to safeguard the extended family’s honor and respectability as well as to prevent children from experiencing their family’s disruption. Scientific literature also highlights that, especially if initiated by a woman, divorce brings shame and social stigma upon the family and children (Savaya & Cohen, 2003). Families generally encourage spouses’ cohabitation despite the occurrence of violence (Abu-Ras, 2007) and for this reason before taking the decision to leave, a woman should be at serious risk;
4. Migration, with the multiple challenges it brings about, results in an increased emotional vulnerability for both men and women (Abraham, 2000a, 2000b). *Feelings of loneliness* for men and *isolation* for women may become unbearable. As clearly evidenced by our findings, the distance from the families of origin that usually act as mediators and role models for the spouses posit immigrant couples at greater risk of segregation. The focus groups analysis show the key role played by the spouses’ families of origin: back in the homeland, the spouses’ parents help maintaining the couple stability, support the husband in running the household and perform a protective function toward the wife and children while similar sources of support are either non-existent or greatly underused in the hosting country. As a result, couple members are left alone to face the many challenges posit by the new context as well as the everyday family duties. Such a condition, characterized by a great emotional load combined with the lack of both social and economic resources, facilitates the onset and perpetration of violent behaviors against women (Hassouneh-Phillips, 2001).

Besides the above mentioned topics, common to all the participants, irrespectively of their country of origin and gender, some specific considerations can be drawn with respect to nationality and gender.

With regards to nationality, Pakistani interviewees not only acknowledge the possible occurrence of violence in intimate relations but also its role as a social regulator, making it a legitimate mean to correct socially unacceptable behaviors. Violence is thus normalized, especially as it serves the need for women’s social control. Men have the specific duty of guardianship over women, they are called to protect and compensate for their frailty and lack of social skills (Ali et al., 2014).

Moreover, women’s isolation as a result of a lack of contacts with the Italian context is a recurrent theme in both Pakistani males’ and females’ interviews. We agree with the literature on the topic in asserting that women’s estrangement from the community and lack of social support in the hosting country might contribute to explain the invisibility of violence that often remains unnoticed and concealed beneath the house’s walls (Abu-Ras, 2000; Kulwicki, Aswad, Carmona, & Ballout, 2010).

Differently from the other participants, Moroccan interviewees show a greater awareness of the risk factors connected to migration in determining the onset and perpetration of domestic violence: economic and work-related difficulties, liberalization of men’s risky or illegal behaviors, traditional gender role patterns that are replicated in the host country with no possibility of being altered are all mentioned as possible causes facilitating the explosion of violence within the couple.

As far as gender differences are concerned, two main topics emerge from the analysis of the focus groups. Women only, irrespectively of their country of origin, report a lack of self-determination and a limited possibility of choice compared to men. This is especially important if we consider that women with low autonomy have been found to be at greater risk for domestic violence. Consistently with the literature on the topic, our female interviewees report the need for women to obey and be subdued to their husbands (Shalabi et al., 2015; Petersen et al., 2003). In this respect, it is also interesting to notice that the issue of arranged marriages, as the expression of the passive acceptance required to Muslim women within the marital couple, was brought up by female participants only.

The distance from the families of origin acquires a slightly different meaning based on gender: while women report a lack of protection and guardianship (especially when problems and couple disagreements degenerate giving rise to violent behaviors), men’s distance from their kin either causes them to feel overwhelmed by family duties and responsibilities or give way to unacceptable, deviant (drug or alcohol abuse, illegal activities) or even violent behaviors. As shown by several studies, drug and alcohol abuse along with unemployment and financial strains (Efe & Ayaz, 2010) increase the likelihood of violent behaviors taking place within the couple.

### Implications for Practice

The topic of violence in immigrant couples posits a series of relevant questions pertaining possible interventions: how can professionals (physicians, psychologists, social workers, law enforcement agencies and other service providers) face this issue?

Along with relevant similarities, the differences between the three ethnic group considered with respect to their attitudes towards domestic violence and gender role expectations suggest the need for both researchers and service providers to also take into account, among other variables, the country of origin of immigrants for not running the risk of equating all Muslims. Actually, it must be underlined that there is no such thing as one Muslim culture, rather, the Muslim world is rich with multiple differences both between as well as within countries (Hamada, 2001). An example of this concerns divorce: contrary to the other two ethnic minorities considered, Moroccan participants view divorce as something that, although undesirable and reproachable, can be accepted.

As extensively discussed above, families of origin regulate couple disputes and are conceived as help-seeking sources with the result that violence keeps being a private “affair”, to be handled within the house’s walls. Such cultural norms regarding family privacy constitute a great barrier to services utilization. Especially if provided by members of hosting country, domestic violence services are regarded as unsafe and culturally inappropriate (Kulwicki et al., 2010). The women in our sample, especially those coming from Pakistan, lack the relevant connections and knowledge about existing services and are isolated from the community life in the hosting country. Promoting connections within immigrant communities, facilitating the relationships between immigrant families and immigrant communities, developing and fostering interventions promoted by immigrant organizations and associations may help view the matter as a public concern rather than a private issue that needs to be resolved within the family.

The development of culturally competent services and the involvement of people sharing the same cultural and religious beliefs of the immigrants seeking help may provide an answer to the feeling of isolation and loneliness both our male and female participants reported, especially in case of couple distress.

Our finding that, when faced with post migration strains and lacking the support of their network, immigrant men might carry out illicit behavior or abuse drugs and alcohol sheds light on an, often underrepresented, problem in migration studies and calls for the need of developing services targeted at men and not exclusively at women. In this perspective, promoting periodic meetings between male immigrants from the same country of origin/ethnic group conducted by leading figures within their community might help men voice their feelings as well as facilitate the development of formal and informal peer support groups, thus reducing their isolation.

In a similar fashion, the outcomes of the present study highlight the importance of providing occasions for meeting and exchange among immigrant women sharing the same cultural background, this will likely multiply their sources of support as well as increase their satisfaction with their lives in the hosting country.

### Limitations

The topic dealt with in the preset study needs to be further investigated within a larger sample in order to assess the reliability and external validity of the topics emerged.

Moreover, the low educational level of the participants involved together with their lack of fluency in Italian might have affected their ability to describe their feelings and thoughts in a more nuanced and richer way, therefore limiting the number and specificity of the themes emerged. Further studies should therefore include participants with different educational background as well as with a higher language proficiency.

The introduction of a quantitative instrument assessing the level of acculturation might also be useful to provide a better interpretation of the participants’ narratives. It might be hypothesized that longer-term residents develop greater levels of assimilation of the hosting culture’s values and representations therefore showing similar attitudes towards couple-related problems to those of the non-immigrant population.

A further limitation might be constituted by the choice of the focus group as the data collection instrument. Focus groups have the advantage of allowing the quick recruiting of a large number of participants at lower costs if compared to individual interviews which also require a greater personal involvement. Moreover, focus groups are particularly suited for gaining an insight of the respondents’ attitudes, beliefs and values which are easily expressed through social interaction. Despite its many advantages, it should be noted that focus groups might also have some limitations, for example that of silencing or reducing the impact of minority opinions. Discussing highly emotionally connoted topics in a group context might lead to stereotypical answers as well as increase the social desirability bias towards both the group members as well as the moderators. The social desirability bias towards the in-group members might be reduced by introducing in-depth individual interviews aimed at further discussing and investigating the topics emerged during the focus groups. Moreover, the social desirability bias towards the interviewers might be curtailed by having one of the two moderators sharing the same cultural background as the participants.
